# Latent profiles identified from psychological test data for people convicted of sexual offences in the UK

**DOI:** 10.1192/bjp.2023.126

**Published:** 2023-12

**Authors:** Steven M. Gillespie, Ian A. Elliott

**Affiliations:** Department of Primary Care and Mental Health, University of Liverpool, Liverpool, UK; His Majesty's Prison and Probation Service, Ministry of Justice, London, UK; and Department of Security and Crime Science, University College London, London, UK

**Keywords:** Mental health services, psychiatry and law, psychological testing, risk assessment, sexual offending.

## Abstract

**Background:**

One size does not fit all in assessment and intervention for people with convictions for sexual offences. Crime scene indicators and risk-related variables have been used to identify distinct clusters of people with convictions for sexual offences, but there is a need for more robust typologies that identify clusters based on psychologically meaningful risk factors that can be targeted in treatment.

**Aims:**

To use robust modelling techniques to identify latent profiles of people with convictions for sexual offences based on indicators of dynamic risk.

**Method:**

Adult male participants, who had been convicted for sexual offences and assessed for eligibility for the prison-based Core Sex Offender Treatment Programme delivered by His Majesty's Prison and Probation Service (UK), were randomly allocated to a test (*n* = 1577: 70.2%) or validation (*n* = 668: 29.8%) data-set. Exploratory factor analysis (EFA) was used to select measures of dynamic risk from psychological test data. EFA indicated four factors, from which six measures were selected for inclusion in latent profile analysis.

**Results:**

Five latent profiles were identified in the test and validation data-sets. These were labelled low psychological impairment, impulsive, distorted thinker, rape preoccupied and child fantasist. Profiles varied in individual characteristics, offence histories, victim preferences and level of risk.

**Conclusions:**

Our findings should be used to guide assessment and intervention practices that are tailored to distinct psychological profiles consistent with principles of risk, need and responsivity.

Sexual violence is an international public health concern that entails a substantial cost to society.^1^ Considerable effort has been invested in assessment and intervention for people convicted of sexual offences, with mixed success.^2^ People who sexually offend have differing motivations, attitudes and beliefs that are associated with their offending behaviour.^3^ These features – termed dynamic risk factors – have been categorised into four broad domains in the Structured Assessment of Risk and Need (SARN):^4,5^ sexual interests, distorted attitudes, socio-affective functioning and self-management. The SARN is one of the most reliable and well-validated frameworks of dynamic risk, and these four domains have been treatment targets on offender behaviour programmes developed by His Majesty's Prison and Probation Service (HMPPS). However, one of the largest barriers to effective assessment and intervention persists in the form of varying profiles of risk and need exhibited by different individuals. In such cases, the ‘one size fits all’ approach is clearly limited.

Given this heterogeneity, it is unsurprising that attempts have been made to categorise people into more homogeneous groups on the basis of offence type (e.g. contact versus online offending), victim age (e.g. prepubescent versus pubescent) or victim familiality.^6^ More sophisticated typologies have employed clinical case files and standardised tests to assign individuals to various categories.^7,8^ However, these models have been criticised on several grounds. Some of the main critiques include the exclusion of incest offenders, the complexity of the classification systems, the time-consuming nature of the classification process and difficulty accounting for so-called ‘cross-over’ offenders who have offended against adults and children.^9,10^ More recently, advanced statistical techniques have been used to allocate people to distinct classes using crime scene indicators,^11,12^ but such typologies are less revealing about the specific changeable (or ‘dynamic’) risk factors that, when targeted in treatment, may be expected to reduce the risk of recidivism.

In this exploratory study, we used latent profile analysis (LPA) to identify distinct subtypes of people convicted of sexual offences based on indicators of dynamic risk from psychological test data. We aimed to build on earlier work, which identified dynamic risk clusters that largely resembled a continuum of severity,^13^ by employing more robust modelling techniques and using a large sample divided into test and validation data-sets. We also examined whether the resulting profiles were distinguishable based on individual characteristics, offence histories and victim preferences.

## Method

### Sample

Participants were part of a data-set of 2394 UK adult males convicted of sexual offences. All participants were assessed between 2003 and 2014 (with 65% of assessments occurring between 2007 and 2011, inclusive) for eligibility for the prison-based version of HMPPS Core Sex Offender Treatment Programme (SOTP). Approximately 97% of the sample was serving a sentence for a conviction between 1996 and 2012, with 77% convicted between 2003 and 2009. Ages ranged between 21 and 84 years (mean 41.8, s.d. = 12.7, median 42 years). Most of the sample was recorded as being in aggregate White ethnic categories (89.8%), with 5.9% classified in aggregate Black ethnic categories, 2.7% in aggregate Asian ethnic categories, 1.3% in aggregate ‘mixed-race’ ethnic categories, 0.2% in aggregate ‘other’ ethnic categories, with a further 0.1% not specified and 4.4% of the data missing. Most of the full sample (*n* = 1526: 68.0%) attended the Core SOTP (rolling or fixed formats), 362 (16.1%) attended a combination of the Core and Extended SOTP, 315 (14.0%) attended the Better Lives Booster (for individuals with intellectual difficulties) and 42 (1.9%) attended the Healthy Sexual Functioning programme (information about Core and Extended SOTP is given in Supplementary Material A, available at https://doi.org/10.1192/bjp.2023.126). Consequently, our sample was heterogeneous regarding executive functioning, sexual interests and victim types.

After removing duplicates and individuals for whom more than 10% of data were missing, a final data-set of 2245 participants remained for analysis. Participants were randomly allocated to a test (*n* = 1577: 70.2%) or validation (*n* = 668: 29.8%) data-set. Results from a simulation study have suggested that a minimum sample size of 500 should be sufficient to accurately identify a correct number of latent profiles.^14^

Ethics approval was not received for this human study because we used existing data collected by HMPPS for the purposes of service evaluation. All adult participants provided written informed consent for their data to be used in research. The project was approved by the Ministry of Justice National Research Committee (granted 5 May 2021).

### Measure selection

The clinical data-set contained pre- and post-programme test scores on 92 scales from 17 psychological measures (see Supplementary Material A for descriptions), along with demographic, intervention and offence characteristics. Only pre-programme test scores were considered for inclusion. One scale was removed because of missing data (30% missing) and two further scales were removed because of perfect or near-perfect positive correlations with other study measures (Supplementary Material A). Any scales that were positively oriented were reverse scored so that higher scores were indicative of greater risk/impairment. Forty scales were removed because of a lack of theoretical support as psychologically meaningful risk factors.^3^

Exploratory factor analysis (EFA) was employed to reduce the number of variables, with the aim of selecting one variable per theoretically plausible risk domain for use in the LPA. Three metrics were used to judge how many factors could plausibly be extracted from the data: (a) parallel analysis; (b) Velicer's minimal average partial (MAP) criterion; and (c) the very simple structure (VSS) criterion.^15^ Parallel analysis revealed that the eigenvalues of seven components exceeded the associated simulated eigenvalue generated from random data. Both the MAP (smallest average squared partial correlation of 0.0129) and the VSS criterions (maximum correlation of 0.79) suggested four components. Since all three tests broadly indicated that additional value is limited beyond four factors (Supplementary Fig. S1, Supplementary Material A), EFA using a varimax rotation and a maximum likelihood solution was used to establish the fit of a four-factor model. Supplementary Table S1 (Supplementary Material A) provides the factor loadings per scale cluster. The root mean square of the residuals (RMSR) was 0.05 (d.f. corrected RMSR = 0.06), χ^2^(524) = 4.2, *P* < 0.001.

The EFA resulted in four classes of scales, approximating three of the four SARN domains of risk:^4^ factor 1, socio-affective and emotional management; factor 2, sexual preoccupation/interests, child specific; factor 3, sexual preoccupation/interests, non-child specific; and factor 4, pro-offending thinking (adult and child). Finally, we constructed correlation matrices for each of the four factors, to ensure that we selected scales for inclusion in the LPA that (a) had a sufficiently high factor loading and (b) had a high average correlation with other scales and thus were a good exemplar of the underlying general construct.

A total of six subscales were selected for inclusion in the LPA: one from the socio-affective and emotional management factor (Impulsive carelessness:^16^ factor loading FL = 0.88, mean *r* (*r*^m^) = 0.61); one from the sexual preoccupation/interests, child-specific factor (Child molest: fantasy:^17^ FL = 0.81, *r*^m^ = 0.53); two from the sexual preoccupation/interests, non-child-specific factor, representing interests (Rape: fantasy:^17^ FL = 0.73, *r*^m^ = 0.43) and preoccupations (Sexual obsession:^17^ FL = 0.58, *r*^m^ = 0.40); and two from the pro-offending thinking factor, one non-child specific (Rape myth acceptance:^18^ FL = 0.74, *r*^m^ = 0.55) and one child specific (Sex with children:^19^ FL = 0.78, *r^m^* = 0.54).

### Socially desirable responding

Given concerns about the tendency towards socially desirable responding, we used the Balanced Inventory of Desirable Responding (BIDR)^20^ to assess the extent to which response bias was problematic in the current sample compared with normative values. Reassuringly, earlier work with a subset of our sample showed that the extent of socially desirable responding was relatively small, and its impact on self-report measures was lower than expected.^21^

### Latent profile analysis

Model-based clustering is based on the theory that data are derived from a mixture of underlying probability distributions.^22^ The most popular approach is the Gaussian mixture model, where each observation is assumed to be distributed as one of *k* multivariate-normal distributions, where *k* is the number of ‘mixture components’ or profiles.^23^ We estimated the optimum number of latent profiles using standardised raw scale scores and estimating profiles as finite mixture models. Data were analysed using R (version 3.5.1 for Windows) and primarily a combination of the ‘tidyLPA’ (version 1.0.8) and ‘mclust’ (version 5.4.6) packages. Gaussian finite mixture models were estimated using the expectation–maximisation (EM) algorithm (starting with the expectation step) for model-based hierarchical agglomerative clustering.^23^

It is recommended that a range of criteria are used for identifying the correct number of profiles.^14^ Although the Bayesian information criterion (BIC)^24^ is used as the default, we also used the Akaike information criterion (AIC),^25^ consistent AIC (CAIC),^26^ sample size adjusted BIC (SABIC)^27^ and integrated complete-data likelihood criterion (ICL)^28^ to judge model estimation. Larger log-likelihood values (BIC, SABIC, AIC, CAIC or ICL) relative to the previous model indicate better fit. Entropy and minimum probability were also assessed,^29^ with values closer to 1 indicative of better fit. Finally, we examined findings from the bootstrap likelihood ratio test (BLRT),^30^ with larger changes in log-likelihood relative to the previous model indicative of better fit. We calculated estimates for one to nine profiles, generated via two models that presumed either equal variances (as opposed to allowing variances to vary) and covariances fixed to zero (Model 1 in tidyLPA) or equal variances and equal covariances (Model 3 in tidyLPA).

### Validity of latent profiles

The replicability of the final profile solution was validated using the validation data-set (30% of the total sample). To establish criterion-related evidence, a series of linear regression models and tests of association were used to examine whether profile allocation was associated with theoretically plausible criminological outcomes, including intelligence, assessed using various versions of the Wechsler Adult Intelligence Scale or Wechsler Abbreviated Scale of Intelligence,^31–33^ age, socially desirable responding, prior convictions (sexual, violent and non-sexual-non-violent), risk level (via the Risk Matrix 2000/S)^34^ and index offence codes that were child specific, related to indecent images of children (IIOC) or female specific. For each outcome, mean differences in scale score between profiles were calculated and plotted alongside Cohen's *d* effect sizes for each pairwise contrast.

## Results

### Socially desirable responding

Scores on the BIDR subscales in the test sample (Self-Deceptive Enhancement: mean 5.9, s.d. = 3.4; Impression Management: mean 6.3, s.d. = 4.0) showed that the extent of socially desirable responding was small and within the range of normative values reported in the BIDR manual (Self-Deceptive Enhancement: mean 7.5, s.d. = 3.2; Impression Management: mean 4.3, s.d. = 3.1).^35^

### Latent profile analysis

Seventy-two cases were removed owing to missingness, and 1505 cases were included in the LPA. BIC and SABIC indicated that Model 3 generated a better relative fit than Model 1 and showed a plateau at five latent profiles (Supplementary Fig. 2, Supplementary Material A). For Model 3, ICL and BLRT were also highest at five latent profiles, and both entropy and minimum probability steeply dropped at five latent profiles. Consequently, a five-profile solution was employed with fixed variances and equal covariances (Model 3: BIC = 21 167.07, ICL = −21 973.86, entropy 0.84).

**Fig. 1:**
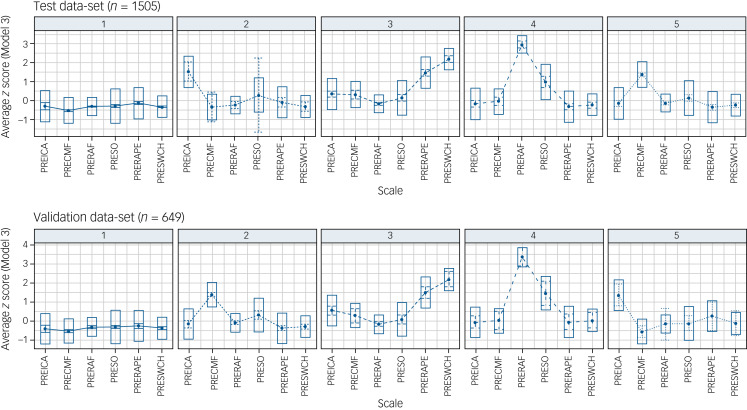
Five-profile solutions with fixed variances and fixed covariances for the test and validation data-sets. PREICA, pre-treatment impulsive carelessness; PRECMF, pre-treatment child molest: fantasy; PRERAF¸ pre-treatment rape: fantasy; PRESO, pre-treatment sexual obsession; PRERAPE, pre-treatment rape myth acceptance; PRESWCH, pre-treatment sex with children. Top row: 1, Low psychological impairment; 2, Impulsive; 3, Distorted thinker; 4, Rape preoccupied; 5, Child fantasist. Bottom row: 1, Low psychological impairment; 2, Child fantasist; 3, Distorted thinker; 4, Rape precoccupied; 5, Impulsive.

The proportion of the overall sample allocated to each profile and aggregate probability values are shown in Table 1. The five profiles (Fig. 1) could be broadly considered to represent: (a) low psychological impairment (LPI); (b) impulsive; (c) distorted thinker; (d) rape preoccupied; and (e) child fantasist. Overall, the mean probability for all latent profile classifications was 0.902 (s.d. = 0.14, minimum 0.401, maximum 1.00) and 81.6% of participants were assigned to a profile with a probability greater than 0.8.

**Table 1:** Proportion of test cases (*n* = 1505) allocated to each profile and aggregate probability values

Latent profile	*n*	%	Probability		
			Minimum	Maximum	Mean (s.d.)
Low psychological impairment	773	51.4	0.492	0.999	0.903 (0.13)
Impulsive	126	8.4	0.428	0.999	0.770 (0.17)
Distorted thinker	186	12.4	0.473	1.000	0.945 (0.11)
Rape preoccupied	122	8.1	0.469	1.000	0.985 (0.06)
Child fantasist	298	19.8	0.393	1.000	0.897 (0.14)

### Solution validation

Our validation data-set generated a very similar five-profile solution. Nineteen cases were removed owing to missingness, and 649 cases were included in the validation LPA. For Model 3, BIC and SABIC plateaued at five profiles, ICL and BLRT were also high at five profiles, and both entropy and minimum probability steeply dropped at five profiles. The Model 3 five-profile solution generated similar profile characteristics and similar group proportions compared to the test sample (Fig. 1). These five profiles could also be broadly considered to represent: (a) LPI (*n* = 323; 49.8%); (b) child fantasist (*n* = 133; 20.5%); (c) distorted thinker (*n* = 83; 12.8%); (d) rape preoccupied (*n* = 42; 6.5%); and (e) impulsive (*n* = 68; 10.5%). Although the proportion of child fantasists was higher in the validation data-set, a χ^2^ association test for the groups derived from the test and validation data-sets was non-significant (χ^2^(1) = 20.0, *P* = 0.22). The overall mean probability for profile allocation was 0.898 (s.d. = 0.14, minimum 0.295, maximum 1.00), with 80% of participants allocated with a probability exceeding 0.80 and a mean probability >0.80 for all five validation profiles.

### Criterion validation

Table 2 shows values for a variety of continuous (numerical) and categorical criminologically relevant variables as a function of profile. For regression analyses, the LPI profile was chosen as the reference against which to compare the other profiles. Results of criterion validation analyses are shown in Table 3. The impulsive profile was associated with significantly lower IQ, and the child fantasist profile with significantly higher IQ, compared with the LPI profile. The distorted thinkers (relative mean difference of +5.4 years) and child fantasists (+6.7 years) were significantly older than those with an LPI profile. Individuals with an impulsive profile had a significantly greater number of prior convictions than those with an LPI profile, whereas those with a distorted thinker or child fantasist profile had significantly fewer prior convictions. Individuals with an impulsive, rape preoccupied or child fantasist profile – but not a distorted thinker profile – all had significantly more sexual convictions than those with an LPI profile. Interestingly, all profiles, on average, were associated with significantly lower socially desirable responding, with large associated effect sizes, suggesting that those with an LPI profile might better be described as ‘socially desirable responders’.

**Table 2:** Means and standard deviations for continuous variables

Variable	Latent profile				
	Low psychological impairment	Impulsive	Distorted thinker	Rape preoccupied	Child fantasist
IQ, mean (s.d.)	98.5 (13.9)	93.7 (12.5)	100 (15.6)	101 (12.1)	107 (14.5)
Age, mean (s.d.)	39.5 (12.2)	38.3 (12.6)	44.9 (14.5)	40.6 (10.4)	46.2 (12.2)
BIDR score, mean (s.d.)	13.7 (6.52)	8.66 (4.9)	9.46 (5.95)	10.7 (6.39)	12 (6.04)
Denial, mean (s.d.)	4.34 (2.21)	4.24 (2.05)	5.04 (2.42)	3.25 (0.72)	3.27 (0.81)
All priors, mean (s.d.)	4.67 (6.15)	6.24 (7.03)	3.66 (4.73)	4.92 (5.42)	2.9 (4.67)
Sexual priors, mean (s.d.)	0.46 (0.91)	0.8 (1.4)	0.57 (1.05)	0.96 (1.32)	0.81 (1.25)
Violent priors, mean (s.d.)	0.37 (1.04)	0.41 (0.99)	0.22 (0.82)	0.36 (0.79)	0.14 (0.78)
Non-sexual-non-violent priors, mean (s.d.)	3.85 (5.65)	5.04 (6.11)	2.86 (4.32)	3.61 (5.03)	1.96 (3.91)
Risk Matrix 2000/S level, *n* (%)					
Low	6 (0.8)	0 (0.0)	0 (0.0)	2 (1.6)	1 (0.3)
Medium	435 (56.4)	55 (43.7)	114 (61.3)	39 (32.0)	152 (51.0)
High	240 (31.0)	45 (35.7)	53 (28.5)	42 (34.4)	94 (31.5)
Very high	91 (11.8)	26 (20.6)	19 (10.2)	39 (32.0)	51 (17.1)
Child-specific index offence, *n* (%)					
Yes	136 (17.6)	20 (15.9)	50 (26.9)	10 (8.2)	84 (28.2)
No	637 (82.4)	106 (84.1)	136 (73.1)	112 (91.8)	214 (71.8)
Indecent images of children (IIOC) index offence, *n* (%)					
Yes	73 (9.4)	13 (10.3)	41 (22.0)	13 (10.7)	93 (31.2)
No	657 (85.0)	103 (81.7)	136 (73.1)	100 (82.0)	195 (65.4)
Indeterminable	43 (5.6)	10 (7.9)	9 (4.8)	9 (7.4)	10 (3.4)
Female-specific index offence, *n* (%)					
Yes	347 (44.9)	56 (44.4)	105 (56.6)	29 (23.8)	154 (51.7)
No	425 (55.1)	70 (55.6)	81 (43.5)	93 (76.2)	144 (48.3)

BIDR, Balanced Inventory of Desirable Responding; priors, prior convictions.

**Table 3:** Results of regression analyses for continuous variables

Variable profile	*R*^2^	*B*	s.e. *B*	*β*	*P*	*d* (*r*)
IQ	0.06					
Impulsive		−4.84	1.53	−0.54***	<0.001	0.19 (0.09)
Distorted thinker		1.90	1.17	0.21	0.104	−0.09 (0.04)
Rape preoccupied		2.80	1.43	0.31*	0.049	−0.10 (0.05)
Child fantasist		8.30	0.98	0.92***	<0.0001	−0.45 (0.22)
Age	0.53					
Impulsive		−1.18	1.24	−0.15	0.344	0.05 (0.02)
Distorted thinker		5.37	1.06	0.68***	<0.0001	−0.27 (0.14)
Rape preoccupied		1.07	1.27	0.14	0.399	−0.05 (0.02)
Child fantasist		6.63	0.88	0.84***	<0.0001	−0.41 (0.20)
All priors	0.03					
Impulsive		1.57	0.55	0.44**	0.004	−0.14 (0.07)
Distorted thinker		−1.01	0.37	−0.28*	0.032	0.11 (0.06)
Rape preoccupied		0.25	0.57	0.07	0.658	−0.02 (0.01)
Child fantasist		−1.77	0.40	−0.49***	<0.0001	0.23 (0.12)
Sexual priors	0.27					
Impulsive		0.34	0.10	0.50**	0.001	−0.17 (0.08)
Distorted thinker		0.12	0.09	0.17	0.193	−0.07 (0.03)
Rape preoccupied		0.50	0.11	0.73***	<0.0001	−0.24 (0.12)
Child fantasist		0.35	0.07	0.51***	<0.0001	−0.24 (0.12)
BIDR score	0.79					
Impulsive		−5.00	0.59	−1.25***	<0.0001	0.44 (0.22)
Distorted thinker		−4.20	0.51	−1.05***	<0.0001	0.43 (0.21)
Rape preoccupied		−2.92	0.61	−0.73***	<0.0001	0.25 (0.12)
Child fantasist		−1.70	0.43	−0.42***	<0.0001	0.21 (0.10)

BIDR, Balanced Inventory of Desirable Responding; priors, prior convictions.

*P < 0.05, **P < 0.01, ***P < 0.001.

Chi-squared tests of association for categorical variables also indicated several group differences. Statistically significant differences in Risk Matrix 2000/S category were found (*χ*^2^(4) = 61.9, *P* < 0.0001, *ϕ* = 0.12), with residuals identifying a higher-than-expected frequency of very high-risk individuals (*z* = 5.01) and fewer medium-risk individuals (*z* = −3.22) with a rape preoccupied profile. There were also fewer than expected very high-risk individuals with an LPI profile (*z* = 2.39).

There were significant associations between profile allocation and proportions having child-specific index offences (*χ*^2^(4) = 33.5, *P* < 0.0001, *ϕ* = 0.15), IIOC index offences (*χ*^2^(4) = 86.9, *P* < 0.0001, *ϕ* = 0.25) and female-specific index offences (*χ*^2^ (4) = 37.4, *P* < 0.0001, *ϕ* = 0.16). The frequency of child-specific index offences in the distorted thinker (*z* = 2.20) and child fantasist (*z* = 3.18) profiles was higher than expected, and lower than expected in the rape preoccupied profile (*z* = −2.94). The frequency of IIOC index offences in the child fantasist (*z* = 6.80) and distorted thinker (*z* = 2.14) profiles was higher than expected, and lower than expected in the LPI profile (*z* = −4.25). The frequency of distorted thinkers (*z* = 2.11) who had female-specific index offences was higher than expected, and lower than expected in the rape preoccupied profile (*z* = −3.66).

### Exploratory analyses

In a *post hoc* set of analyses, we explored the effects of socially desirable responding on the classification of those with an LPI profile. We isolated the cases assigned to the LPI profile in the test data-set and corrected for socially desirable response bias using a statistical technique devised by Saunders.^36^ We then repeated the LPA. The results suggest that approximately one-third of those with an LPI profile might be more appropriately allocated to one of the alternative profiles (Supplementary Material B).

## Discussion

In a pre-treatment sample of people convicted for sexual offences in the UK, we used psychological test measures, indexing three of the four SARN domains of dynamic risk,^4^ to identify five latent profiles in test (*n* = 1577) and validation (*n* = 668) samples. The first profile was characterised by a relative lack of psychological impairments, accounted for roughly half of the overall test sample and showed low scores across all test measures. People with this profile showed few identifying individual or criminological characteristics, except for higher image management and/or self-deceptive enhancement, and follow-up tests adjusting for socially desirable responding suggested that at least one-third of this group may be better allocated to an alternative profile. Attempts to manage people in this profile may therefore be compromised by response bias tendencies.

The second profile was termed impulsive and accounted for approximately 8.4% of the test sample. These individuals were characterised by heightened impulsive carelessness, had relatively low IQ and a relatively high number of overall convictions. People allocated to this profile appear to conform to a more ‘generalist’ antisocial pattern of offending^37^ that approximates impulsive or generally antisocial subtypes identified by others.^9,38^

Approximately 12.4% of the test sample were allocated to a distorted thinker profile and were characterised by relatively high scores for rape myth acceptance and distorted thinking about children and sex. Distorted thinkers tended to be older, had fewer overall convictions, with a relatively high frequency of index offences that were child specific, female specific or related to IIOC, consistent with a preference for younger victims despite elevated scores for rape myth acceptance.

The final two profiles were termed rape preoccupied and child fantasist and accounted for 8.1 and 19.8% of the test sample respectively. People with these profiles showed more specific areas of risk and need related to deviant sexual preferences and preoccupations with sex. The rape preoccupied group accounted for the smallest proportion of the test sample and included individuals who showed particularly elevated scores for rape fantasies and non-child-specific obsessive thinking about sex. People allocated to a rape preoccupied profile had a relatively high number of previous sexual offences, with a relatively high frequency of very high-risk individuals but fewer medium-risk, and relatively few people with child-specific and female-specific index offences. People in this profile appear to show a preference for adult victims but offended less preferentially against female victims.

Child fantasists showed a specific elevation for child and sex fantasies, and tended to be older and to have higher IQs relative to the LPI profile. This profile included a disproportionately high frequency of child-specific and IIOC index offences and a much less extensive overall criminal history. People with this profile may conform to a more high-functioning, preferentially paedophilic pattern of offending,^37^ with a history of IIOC offences being a stronger diagnostic indicator of paedophilia than contact sexual offending.^39^

### Clinical implications

Our findings have clear implications for assessment and treatment, and can be considered in the context of SARN domains of dynamic risk, and principles of risk, need and responsivity.^40^ For example, individuals with an impulsive profile may benefit from offending behaviour programmes designed for more generally antisocial and violent offenders, and interventions focused on self-regulation and managing emotions.^41^ This group would benefit minimally from interventions focused on distorted thinking about sex, sexual fantasising or unusual sexual interests. In contrast, those with a distorted thinker profile may benefit from programmes that address distorted thinking patterns related to sex with adults and/or children, but may benefit less, on average, from interventions targeting sexual preoccupation/interests or socio-affective function and emotion management. Individuals with the rape preoccupied profile presented with high levels of both risk and need and should be prioritised for more intensive treatment focused on reducing sexual fantasising and sexual preoccupation. Rape preoccupied individuals who are high risk and show pronounced sexual preoccupations or paraphilic sexual interests may additionally benefit from medication to reduce sexual arousal.^42^ These medications tend to include anti-androgens, selective serotonin reuptake inhibitors and gonadotropin-releasing hormone analogues.^42^ Although there is a lack of robust evidence to support the medical management of sexual arousal, some degree of success has been reported.^43^ Finally, people with a child fantasist profile, where there was a relatively high frequency of IIOC index offences, but few distorted attitudes about children and sex and low sexual preoccupation, may benefit from elements of healthy sex interventions^44^ and psychoeducation about the consequences of IIOC.^45^ However, more robust, long-term outcome studies are needed to understand the potential benefits of these approaches in prisons and in the community.

### Strengths and limitations

Our work has several strengths, including a large, heterogeneous sample, data on intelligence, the number and type of previous convictions, and information about level of risk and index offence codes. However, our results are nonetheless subject to some limitations. First, factor analyses revealed that the psychological test data indexed three of the four domains of risk identified by Thornton,^4^ but did not yield an antisocial component. The inclusion of measures of antisocial personality pathology and psychopathic tendencies would add to the richness of the latent profiles. Second, the LPAs relied solely on data collected using self-reports and are therefore subject to obvious limitations, including socially desirable responding. Future work should include alternative measurements, including clinical checklists and indices of physiological arousal (e.g. penile tumescence to sexual stimuli). Third, information about victim type was also only available for index offences, and we do not know the proportion of individuals in each profile who had prior convictions involving child victims, female victims or use of IIOC. Fourth, profiles derived using LPA might not represent true profiles that exist in the target population. Superfluous classes can be identified owing to non-normality of the data, non-linear relationships between the indicator variables or a misspecification of the model.^46^ The interpretation of profiles is also subjective. Although there are no clear rules on how to make sense of profiles, we followed reviews of best practice and considered the balance of the indicators and the context of the relevant literature.^47^

### Future research

Future work should aim to authenticate the five identified latent profiles within and outside the UK and examine differences between profiles in responsivity to different interventions (including pharmacological interventions where indicated).

## Supporting information

Gillespie and Elliott supplementary material 1 Gillespie and Elliott supplementary material

Gillespie and Elliott supplementary material 2 Gillespie and Elliott supplementary material

## Data Availability

The data that support the findings of this study are available from Ministry of Justice, UK. Restrictions apply to the availability of these data, which were used under licence for this study.
