# Supplementary material for: Latent profiles identified from psychological test data for people convicted of sexual offences in the UK
Source: Br J Psychiatry. 2023 Dec;223(6):555–61. doi: 10.1192/bjp.2023.126 (PMC10727912; doi:10.1192/bjp.2023.126)
Supplement: Gillespie and Elliott supplementary material 1 — Gillespie and Elliott supplementary material [file S0007125023001265sup001.docx]

**Supplemental Material A**

**Core and Extended versions of the Sex Offender Treatment Programme (SOTP)**

The Core SOTP was developed by His Majesty’s Prison and Probation Service with the aim of helping individuals to develop the necessary self-management skills and motivation to avoid committing new offences in the future. It emphasised treatment as a collaborative effort, and included cognitive restructuring, modelling, and positive reinforcement (Beech, Oliver, Fisher, and Beckett, 2005). The average treatment dose of the Core SOTP was reported to be 180 hours (Beech & Mann, 2002). Where a person was assessed as having additional treatment needs that were not targeted during the Core SOTP, they also undertook a second stage of treatment known as the Extended SOTP (Beech et al.,2005). The goal of the extended programme was to help individuals to identify and challenge patterns of dysfunctional thinking, improve emotional management and relationship skills, address deviant sexual fantasies and arousal, and to understand how these motivations, attitudes, and beliefs may have contributed to their offending behaviour (Mann, 1999).

**List of assessments from which scales were drawn.**

The battery of assessments administered to SOTP participants was set out in the SOTP Psychometric Assessment Manual (Version 4). Each measure is described below along with, wherever possible, the reported Cronbach's alpha for internal consistency with a subset of the target population and the size of the criminal justice subset on which validation was conducted (as per the assessment manual). One scale (emotional loneliness) was removed due to missing data (30% missing), and two further scales were removed due to: (1) a perfect positive correlation between the MSI Sado-masochism related to rape scale (retained, due to adult victim focus) and the MSI Sado-masochism related to paraphilia (removed), and (2) a near-perfect negative correlation between negative problem orientation (retained, due to representing a deficient problem orientation) and positive problem orientation (removed).

The Personality Questionnaire is a 36-item measure of a variety of socio-affective constructs adapted from three separate measures: (1) the Short Self-esteem Scale (Thornton et al., 2004; Webster et al., 2007); (2) Eysenck Impulsivity Scale (Eysenck & Eysenck, 1978); The Dissipation-Rumination Scale (Capara, 1986). The self-esteem scale measures self-worth and shame (α = 0.84, *n* = 1,847). The impulsivity scale reflects a tendency to act without thinking about long-term consequences (α = 0.84, *n* = 1,835). The ruminations scale reflects a tendency to ruminate angrily and bear grudges (α = 0.79, *n* = 1,770).

The Interpersonal Reactivity Index (IRI: Davis, 1980) is a 28-item measure of the cognitive and emotional components of empathy. The IRI comprises four sub-scales: fantasy (α = 0.77), empathic concern (α = 0.72), perspective taking (α = 0.72), and personal distress (*a* = 0.74). Higher fantasy scores indicate greater identification with fictional characters. Higher empathic concern scores indicate greater levels of warmth, compassion, and concern for those in trouble or distress. Higher perspective taking scores indicate a greater ability to see others’ points of view. Higher personal distress scores indicate a greater tendency to become anxious or to experience other negative emotions when others are in trouble.

The Locus of Control Scale (Levenson, 1972; 1974) is an 18-item scale that assesses the extent to which a respondent believes what happens to them is determined by external factors or whether they have control over their experiences (α = 0.79, *n* = 1,883).

The Emotional Control Questionnaire (ECQ: Roger & Najarian, 1989) is a 56-item scale, which measures emotional response style. The ECQ comprises four subscales: Rehearsal (α = 0.86, n = 244), Emotional inhibition (α = 0.77, *n* = 244), Aggression control (α = 0.81, *n* = 244), and Benign Control (α = 0.79, *n* = 244). Higher rehearsal scores indicate a greater tendency to ruminate about emotionally upsetting events. Higher emotional inhibition scores indicate a lesser tendency to express emotion. Higher aggression control scores reflect a greater degree of inhibition of feelings of hostility or aggression. Higher benign control scores reflect a lower tendency to act before thinking (equivalent to impulsiveness).

The Relationship Style Questionnaire (Dutton et al., 1994) is a 30-item self-report questionnaire measuring four attachment patterns: Secure (α = 0.41, *n* = 40), Fearful, Pre-occupied, and Dismissing (α = 0.71, *n* = 40). Alpha values were not available for the Fearful and Pre-occupied scales. It was noted in the manual that internal consistencies for the RSQ subscale scores are variable and low due to the combination of two orthogonal dimensions (a “self model” and an “other model”). Individuals demonstrating a secure pattern are confident and comfortable with intimacy in their close relationships. Individuals demonstrating a fearful pattern desire social contact and intimacy but experience pervasive interpersonal distrust and fear of rejection. Individuals demonstrating a pre-occupied pattern seek to gain their attachment figure’s approval to validate their sense of self-worth. Individuals demonstrating a dismissing pattern maintain a positive self-image by defensively downplaying the importance of attachment needs and maintaining emotional distance in their relationships.

The Balanced Inventory of Desirable Responding is a 40-item self-report questionnaire, which measures the tendency to give socially desirable responses (Paulhus, 1984; Mathie & Wakeling, 2011). The PDS contains two subscales: Self-Deception (α = 0.72, *n* = 603) and Impression Management (α = 0.82-0.84, *n* = 603). The self-deception subscale represents an unconscious bias related to narcissism and is designed to assess defensiveness to personal threats and positively biased responding that the respondent believes to be true. The impression management scale is designed to measure responding that is guided by a desire to create a favourable impression on others.

The Social Problem-Solving Inventory Revised (D'Zurilla et al., 2002) is a 52-item self-report measure that assesses one’s ability to resolve problems in everyday living. The SPSI-R comprises five subscales: Positive Problem Orientation (α = 0.73, *n* = 499), Negative Problem Orientation (α = 0.92, *n* = 499), Rational Problem Solving (α = 0.95, *n* = 499), Impulsivity/Carelessness Style (α = 0.91, *n* = 499) and Avoidance Style (α = 0.82, *n* = 499). The total SPSI-R score (α = 0.86, *n* = 499) provides a global indicator of one’s social problem-solving ability. The positive problem orientation scale measures the tendency to see a problem as a ‘challenge’ rather than a threat, believe that problems can be solved, and strive to solve problems rather than avoid them. The negative problem orientation scale measures the tendency to view a problem as a significant threat to well-being, doubt their ability to solve problems, and become upset and frustrated when faced with problems. The rational problem-solving scale measures the tendency to gather facts and information about problems carefully, set realistic goals, generate alternative solutions, anticipate consequences, compare possible alternatives, and choose a solution whilst evaluating the outcome. The impulsive/carelessness style scale measures the tendency to consider few solution alternatives, act impulsively when making decisions, look at alternatives and consequences only quickly, and monitor outcomes inadequately. The avoidance style scale measures the tendency to avoid problems, not confront them, and put off solving problems for as long as possible.

The Sex Offence Attitudes Questionnaire (Hogue, 1994) is a 50-item Sex Offence Attitudes Questionnaire. It is comprised of six subscales relating to levels of cognitive denial, distortion, and minimisation. Subscales are: (1) denial of repetition, that their offence is likely to be repeated (α = 0.74, *n* = 1,950); (2) denial of premeditation, that their offence was purely spontaneous (α = 0.78, *n* = 1,955); (3) denial of harm, that their offence had little consequence for the victim or was just as consequential for the perpetrator (α = 0.85, *n* = 1,924); (4) denial of offence, the maintenance of innocence (α = 0.49, *n* = 1,956); (5) denial of responsibility, that some level of blame for the offence should be borne by the victim or others (α = 0.84, *n* = 1,931); and (6) denial of control, that the offence was primarily due to overwhelming emotional factors (e.g., stress, anger, frustration, or mental health) (α = 0.73, *n* = 1,920). Subscales scores are summed to produce a total minimisation score (α = 0.86, *n* = 1,777).

The Opinions Questionnaire is a 67-item questionnaire comprising six different scales that reflect three separate domains. The domains are attitudes and beliefs that support the sexual abuse of children (Marshall, 1995; Mann et al., 2007), attitudes and beliefs that support the sexual abuse of women (Burt, 1980; Hanson et al., 1994), and openness/emotional congruence with women and men (developed by HMPPS). There are six scales: sex with children is justifiable (α = 0.95, *n* = 1,888); women are deceitful (α = 0.79, *n* = 1,905); entitlement to sex (α = 0.65, *n* = 1,873); rape myth acceptance (α = .83, *n* = 1,851); openness to women (α = 0.87, *n* = 1,909); and openness to men (α = 0.86, *n* = 1,917). The sex with children is justifiable scale measures a greater tendency to endorse justifications for having sex with children. The women are deceitful scale measures the tendency to believe women are untruthful, have malicious intentions, and cannot be trusted. The entitlement to sex scale measures the tendency to believe that men’s (particularly the respondent’s) sexual needs take priority over the sexual needs of others and should be obliged above their own interests. The rape myth acceptance scale measures the tendency to endorse minimizations, rationalisations, and excuses for the sexual assault of women. The openness to women scale measures the respondent’s belief that they can have and enjoy emotionally intimate relationships with women. The openness to men scale measures the respondent’s belief that they can have and enjoy emotionally intimate relationships with men.

The Children and Sex Questionnaire (CSQ: Beckett, 1987) is an 87-item questionnaire that measures respondents’ attitudes, feelings, and thoughts about children and sex. These 30 items are clustered into two sub-scales: (1) cognitive distortions and (2) emotional congruence. Items are summed to produce a total scale score. Cohen's alpha was not reported for the CSQ. Higher scores on the cognitive distortions scale indicate that the respondent utilizes a high level of distortions about children and sex. Higher scores on the emotional congruence scale indicate that the respondent shows high congruence and identification with children.

The Hypermasculnity Inventory (Mosher & Sirken, 1984) is a 30-item forced-choice questionnaire developed to measure a macho personality constellation consisting of three components: (1) callous sex attitudes towards women (α = 0.79, *n* = 135); (2) violence as manly (α = 0.79, *n* = 135); and (3) danger as exciting (α = 0.71, *n* = 135). The callous sex attitudes scale reflects attitudes that sexual intercourse with women establishes masculine power and female submission and is to be achieved without empathic concern for the female or her experience. The violence as manly scale reflects attitudes held by some men that violent aggression, verbal or physical, is acceptable or a preferable expression of power and dominance toward other men. The danger as exciting scale reflects attitudes that survival in dangerous situations is a manly display of masculine power over the dangerous environment.

The Multiphasic Sex Inventory (MSI: Nichols & Molinder, 1984) is a 300-item questionnaire measuring sexual behaviour. The instrument is divided into a number of subscales: (1) Social and Sexual Desirability Scale; (2) Sexual Obsessions Scale, measuring the individual’s preoccupation with sex; (3) Lie Child Molest/Rape Scale, measuring the tendency to use denial and dishonesty to defend deviant sexual activity; (4) Cognitive Distortion/Immaturity Scale, measuring cognitive distortions that may facilitate acting on sexually deviant impulses and feelings of lack of accountability; (5) Justifications Index, measuring the degree to which the offender attempts to justify his sexual offence behaviour; (6) Treatment Index; (7) Child Molest Scale (Fantasy, Cruising/Grooming, Sexual Assault, Aggravated Assault, Incest Type), measuring the extent to which the individual has (or might intend to) manipulate and coerce a child into sexual behaviours; (8) Rape Scale (Fantasy, Cruising, Sexual Assault, Aggravated Assault, Sado-Masochism), measuring the extent to which the individual has (or might intend to) manipulate and coerce non-consenting adults into sexual behaviours; (9) Exhibitionism Scale (Fantasy, Cruising, Sexual Assault, Advanced Assault), measuring sexual behaviours for those with an interest in exposing themselves to non-consenting persons; (10) Paraphilia Scale (Fetish, Voyeurism, Obscene, Bondage /Discipline, Sado-Masochism), measuring interests in uncommon sexual practices; (11) Sex Dysfunction scale (Sexual Inadequacies, Premature Ejaculation, Physical Disabilities, Impotence); (12) Sex Knowledge and Beliefs Scale. See MSI manual for details of scales and the psychometric properties of the tool.

The Relapse Prevention Interview (RPI: Beckett et al., 1997) is an 18-item interview that elicits a respondent’s: (1) recognition of lapse cues (α = 0.85, *n* = 1,307), such as events, situations, emotions, thoughts, and fantasies; (2) possession of coping skills and strategies (α = 0.85, *n* = 1,462), and acceptance of future risk/likelihood of relapse (α = 0.80, *n* = 1,462).

The UCLA Emotional Loneliness Questionnaire (EL: Russell et al., 1980) is a 19-item questionnaire that indicates the extent to which respondents believe they had meaningful relationships, had people close to them or were lonely in the 6 months preceding their assessment (α = 0.95, *n* = 1,330).

**Table S1**

*Factor loadings from exploratory factor analysis of 37 candidate variables*

| Scale | ML1 | ML2 | ML3 | ML4 | Class |
| --- | --- | --- | --- | --- | --- |
| Impulsivity | 0.81 | -0.01 | 0.05 | 0.11 | IMP |
| Denial of harm | 0.07 | -0.05 | -0.08 | 0.65 | OSA |
| Deceitful women | 0.42 | 0.01 | 0.01 | 0.60 | OSA |
| Sex with children | 0.10 | 0.17 | 0.01 | 0.78 | OSA |
| Rape myth acceptance | 0.26 | -0.06 | 0.02 | 0.74 | OSA |
| Sexual entitlement | 0.33 | 0.13 | 0.19 | 0.59 | OSA |
| Cognitive distortions | 0.12 | 0.10 | 0.03 | 0.76 | OSA |
| Emotional congruence with children | 0.14 | 0.19 | 0.08 | 0.39 | OSA |
| Callous sexual attitudes | 0.24 | -0.11 | 0.15 | 0.23 | OSA |
| Negative problem orientation | 0.78 | 0.08 | 0.06 | 0.15 | PRS |
| Impulsive carelessness | 0.88 | 0.04 | 0.05 | 0.15 | IMP |
| Social/sexual desirability | 0.12 | 0.12 | 0.17 | 0.05 | OTH |
| Sexual obsession | 0.33 | 0.30 | 0.58 | 0.11 | SEX |
| Treatment attitudes | 0.25 | 0.47 | 0.32 | -0.16 | OTH |
| Child molest: fantasy | 0.02 | 0.81 | 0.08 | 0.07 | SIIC |
| Child molest: cruising/grooming | 0.06 | 0.75 | 0.12 | 0.15 | SIIC |
| Child molest: sexual assault | 0.05 | 0.86 | -0.09 | 0.02 | SIIC |
| Child molest: aggravated assault | 0.01 | 0.71 | -0.01 | 0.01 | SIIC |
| Child molest: incest | 0.06 | 0.46 | 0.08 | 0.04 | SIIC |
| Child molest: gender girl | 0.10 | 0.64 | 0.02 | 0.03 | SIIC |
| Child molest: gender boy | 0.01 | 0.52 | 0.03 | 0.07 | SIIC |
| Rape: fantasy | 0.01 | 0.04 | 0.73 | -0.03 | SEX |
| Rape: cruising | -0.03 | -0.05 | 0.69 | -0.09 | SEX |
| Rape: aggravated assault | -0.03 | -0.19 | 0.53 | -0.17 | SEX |
| Rape: sadomasochism | -0.01 | -0.05 | 0.69 | -0.01 | SEX |
| Exhibitionism: fantasy | 0.05 | 0.15 | 0.49 | 0.11 | PARA |
| Exhibitionism: cruising | -0.01 | 0.34 | 0.42 | 0.05 | PARA |
| Exhibitionism: advanced assault | 0.01 | 0.07 | 0.43 | 0.02 | PARA |
| Paraphilias: fetish | 0.03 | 0.21 | 0.57 | 0.07 | PARA |
| Paraphilias: voyeurism | 0.15 | 0.19 | 0.67 | 0.09 | PARA |
| Paraphilias: obscene calls | 0.09 | -0.01 | 0.43 | 0.06 | PARA |
| Sexual inadequacy | 0.27 | 0.22 | 0.15 | 0.12 | OTH |
| Marriage development scale | 0.11 | -0.08 | 0.34 | 0.07 | OTH |
| Benign control (reversed) | 0.81 | 0.00 | 0.07 | 0.13 | IMP |
| Openness to women (reversed) | 0.43 | 0.18 | 0.06 | 0.18 | OSA |
| Openness to men (reversed) | 0.48 | 0.13 | 0.07 | 0.22 | OSA |
| Rational problem solving (reversed) | 0.64 | 0.02 | 0.01 | 0.12 | PRS |

**Figure S1**

*The PA, VSS, and MPA findings for number of factors to be used in the EFA*


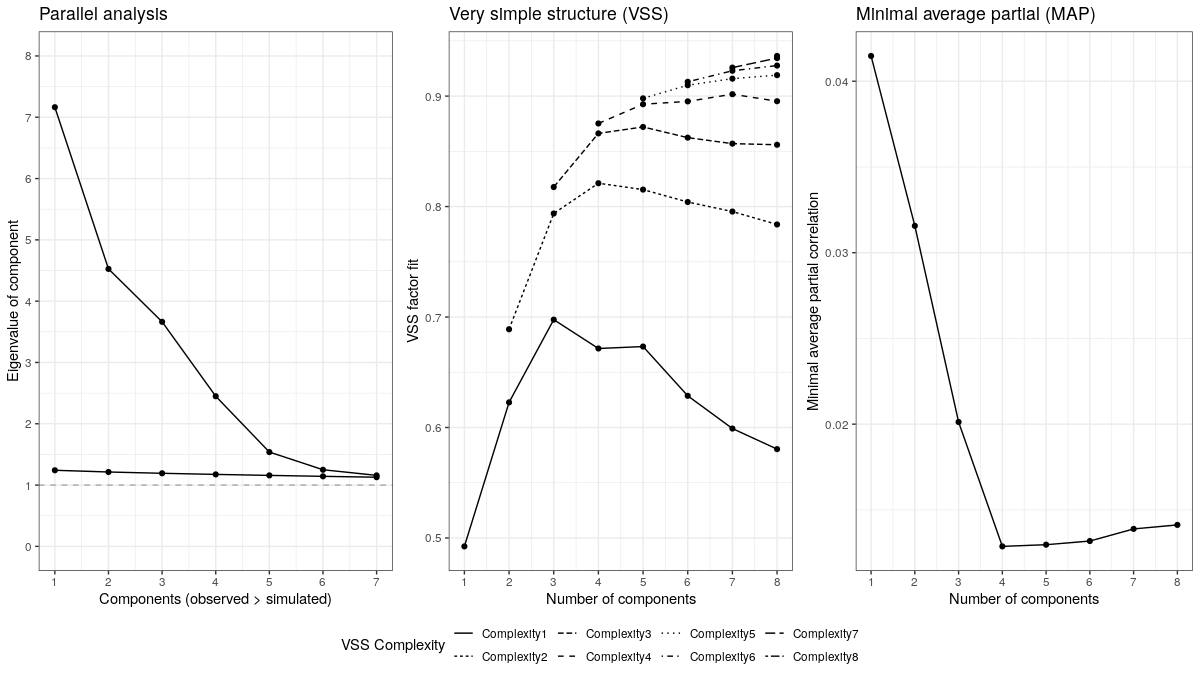


**Figure S2**

*Results of six information criteria for number of profiles to be extracted*


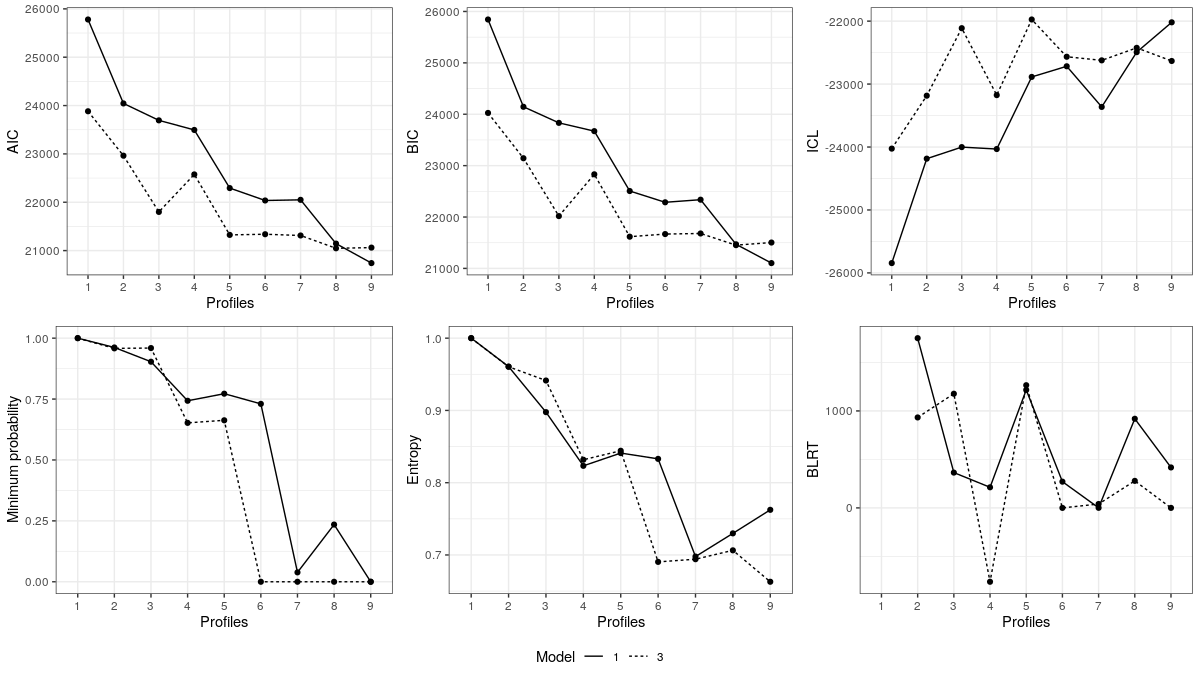


**Supplemental References**

Beckett, R. C. (1987). Children and Sex Questionnaire. Unpublished.

Beckett, R. C., Fisher, D., Mann, R., and Thornton, D. (1997). The Relapse Prevention Questionnaire and Interview. In H. Eldridge (Ed.), *Therapists Guide for Maintaining Change: Relapse Prevention Manual for Adult Male Perpetrators of Child Sexual Abuse*. California, US: Sage Publications.

Beech, A. R., & Mann, R. E. (2002). Recent developments in the treatment of sexual offenders. In J. McGuire (Ed.). *Offender rehabilitation: effective programs and policies to reduce reoffending* (pp. 259-288). Chichester, U.K.: Wiley.

Beech, A., Oliver, C., Fisher, D., & Beckett, R. (2005). STEP 4: *The Sex Offender Treatment Programme in prison: Addressing the offending behaviour of rapists and sexual murderers.* London, U.K.: HM Prison and Probation Service.

Burt, M. R. (1980). Cultural myths and supports for rape. *Journal of Personality and Social Psychology, 38*(2), 217-230.

Capara, G. V. (1986). Indications of aggression: The Dissipation-Rumination Scale. *Personality and Individual Differences, 7*, 763-769.

Davis, M. H. (1980). A multi-dimensional approach to individual differences in empathy. *JSAS Catalogue of Selected Documents in Psychology, 10*, 85-100.

Dutton, D. G., Saunders, K., Starzomski, A., & Bartholomew, K. (1994). Intimacy-anger and insecure attachment as precursors of abuse in intimate relationships. *Journal of Applied Social Psychology, 24*(15), 1367-87.

D’Zurilla, T. J., Nezu, A. M., & Maydeu-Olivares, A. (2002). Social Problem-Solving Inventory–Revised. Technical Manual. MHS.

Eysenck, S. B. G., and Eysenck, H. J. (1978). Impulsivity and venturesomeness: Their place in a dimensional system of personality description. *Psychological Reports, 43*, 1247-1255.

Hanson, R. K., Gizzarelli, R., and Scott, H. (1994). The attitudes of incest offenders: Sexual entitlement and acceptance with children. *Criminal Justice and Behavior, 21*(2), 187-202.

Hogue, T. E. (1994). The Sex Offence Information Questionnaire: The development of a self-report measure of offence related denial in sex offenders. Unpublished PhD thesis.

Levenson, H. (1972). Distinctions within the concept of internal-external control: Development of a new scale. *Proceedings of the 80th Annual Convention of American Psychological Association, 7*, 259-260.

Levenson, H. (1974). Multidimensional locus of control in prison inmates. *Personality and Social Psychology Bulletin, 1*, 354-356.

Mann, R.E. (1999). The sex offender treatment programme HM Prison Service England & Wales. In Hofling, S., Drewes, D. & Epple-Waigel, I. (Eds.), *Auftrag Pravention: Offensive gegen sexuellen kindesmibbrauch* (pp. 346-352). Munich, Germany: Atwerb-Verlag KG Publikation.

Mann, R., Webster, S., Wakeling, H., & Marshall, W. (2007). The measurement and influence of child sexual abuse supportive beliefs. *Psychology, Crime & Law, 13*(5), 443-458.

Marshall, W. L. (1995). Sex with Children is Justifiable Questionnaire. Unpublished.

Mosher, D. L. & Sirken, M. (1984). Measuring a macho personality constellation. *Journal of Research in Personality, 18*, 150-163.

Nichols, H.R. & Molinder, I. (1984). Multiphasic Sex Inventory. A Test to assess the characteristics of the sexual offender. *Nichols and Molinder Assessments.*

Paulhus, D.L. (1984). Two component models of socially desirable responding. *Journal of Personality and Social Psychology, 46*(3), 598-609.

Mathie, N. L., & Wakeling, H. C. (2011). Assessing socially desirable responding and its impact on self-report measures among sexual offenders. *Psychology, Crime & Law, 17*(3), 215-237.

Roger, D., and Najarian, B. (1989). The Construction and Validation of a New Scale for Measuring Emotion Control. *Personality & Individual Differences, 10*(8), 845-853.

Russell, D., Peplan, C. A., and Cutrona, C. A. (1980). The revised UCLA Loneliness Scale: Concurrent and discriminant validity evidence. *Journal of Personality and Social Psychology, 39*, 472-480.

Thornton, D., Beech, A., & Marshall, W. L. (2004). Pre-treatment self-esteem and posttreatment sexual recidivism. *International Journal of Offender Therapy and Comparative Criminology, 48*, 587-599.

Webster, S. D., Mann, R. E., Thornton, D., & Wakeling, H. C. (2007). Further validation of the short self‐esteem scale with sexual offenders. *Legal and Criminological Psychology, 12*(2), 207-216.
