# Supplementary material for: Latent profiles identified from psychological test data for people convicted of sexual offences in the UK
Source: Br J Psychiatry. 2023 Dec;223(6):555–61. doi: 10.1192/bjp.2023.126 (PMC10727912; doi:10.1192/bjp.2023.126)
Supplement: Gillespie and Elliott supplementary material 2 — Gillespie and Elliott supplementary material [file S0007125023001265sup002.docx]

**Supplemental Material B**

**Correction procedure for socially desirable responding**

Consistent with the approach outlined by Saunders (1991), the regression coefficient for predicting the unadjusted score for each measure from the offender’s score on the response validity measure is derived for each comparison group using the simple regression formula: Y = a + (b)(x), where Y is the unadjusted score, (x) is the score on the BIDR, and (b) is the unstandardised regression coefficient. This regression coefficient provides a coefficient by which scores on a measure increase or decrease, within each group, for every 1 unit increase in social desirability (i.e., the effect of increasing levels of socially desirable responding on a group’s scores on a measure). The adjusted score (Y1) is calculated by multiplying the BIDR score by the regression coefficient and subtracting this from the unadjusted score: Y1 = Y – (b)(x).

Metrics (AIC, BIC, ICL, minimum probability, entropy, BLRT) all indicated a five-factor solution with equal variances and covariances fixed to zero (Model 1: BIC = 11,715.92, ICL = -11781.76, entropy = 0.94, minimum probability = .80). The resulting five profiles were slightly different to the main analysis and constituted: (1) high rape interest (97: 12.6%); (2) distorted thinkers (102: 13.2%); (3) low psychological deficits (497: 64.5%); (4) rape preoccupied (14: 1.8%); and (5) child fantasist (61: 7.9%). This suggests that perhaps, at least, approximately one-third of our original low-psychological deficit group could be appropriately allocated to one of the existing profiles.

**References**

Saunders DG. Procedures for adjusting self-reports of violence for social desirability bias. Journal of Interpersonal Violence. 1991; 6(3): 336-44.
